# Characterization of the specific interaction between the DNA aptamer sgc8c and protein tyrosine kinase-7 receptors at the surface of T-cells by biosensing AFM

**DOI:** 10.1007/s00216-017-0238-5

**Published:** 2017-02-22

**Authors:** Michael Leitner, Alexandra Poturnayova, Constanze Lamprecht, Sabine Weich, Maja Snejdarkova, Ivana Karpisova, Tibor Hianik, Andreas Ebner

**Affiliations:** 1 Institute of Biophysics, Johannes Kepler University Linz, Gruberstrasse 40, 4020 Linz, Austria; 2 Faculty of Mathematics, Physics, and Informatics, Comenius University, Mlynska dolina F1, 842 48 Bratislava, Slovakia; 3 Institute of Biochemistry and Animal Genetics, Slovak Academy of Sciences, Moyzesova 61, 900 28 Ivanka pri Dunaji, Slovakia

**Keywords:** DNA aptamer, PTK7, T-cell, Single molecule force spectroscopy, Energy landscape, Molecular recognition, Recognition imaging

## Abstract

We studied the interaction of the specific DNA aptamer sgc8c immobilized at the AFM tip with its corresponding receptor, the protein tyrosine kinase-7 (PTK7) embedded in the membrane of acute lymphoblastic leukemia (ALL) cells (Jurkat T-cells). Performing single molecule force spectroscopy (SMFS) experiments, we showed that the aptamer sgc8c bound with high probability (38.3 ± 7.48%) and high specificity to PTK7, as demonstrated by receptor blocking experiments and through comparison with the binding behavior of a nonspecific aptamer. The determined kinetic off-rate (k_off_ = 5.16 s^−1^) indicates low dissociation of the sgc8c–PTK7 complex. In addition to the pulling force experiments, simultaneous topography and recognition imaging (TREC) experiments using AFM tips functionalized with sgc8c aptamers were realized on the outer regions surface of surface-immobilized Jurkat cells for the first time. This allowed determination of the distribution of PTK7 without any labeling and at near physiological conditions. As a result, we could show a homogeneous distribution of PTK7 molecules on the outer regions of ALL cells with a surface density of 325 ± 12 PTK7 receptors (or small receptor clusters) per μm^2^.

**Graphical Abstract:**
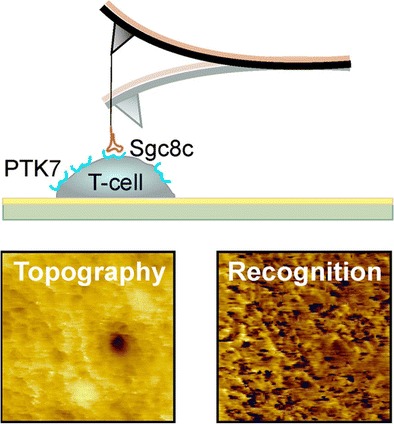
The specific interaction of the DNA aptamer sgc8c and protein tyrosine kinase-7 (PTK7) on acute lymphoblastic leukemia (ALL) cells was characterized. AFM based single molecule force spectroscopy (SMFS) yielded a kinetic off-rate of 5.16 s^−1^ of the complex. Simultaneous topography and recognition imaging (TREC) revealed a PTK7 density of 325 ± 12 molecules or clusters per μm^2^ in the cell membrane

The specific interaction of the DNA aptamer sgc8c and protein tyrosine kinase-7 (PTK7) on acute lymphoblastic leukemia (ALL) cells was characterized. AFM based single molecule force spectroscopy (SMFS) yielded a kinetic off-rate of 5.16 s^−1^ of the complex. Simultaneous topography and recognition imaging (TREC) revealed a PTK7 density of 325 ± 12 molecules or clusters per μm^2^ in the cell membrane

## Introduction

Cancer is a major societal challenge and its detection and identification at the earliest stage are crucial for efficient and successful treatment. Acute lymphoblastic leukemia (ALL) is a common type of blood cancer. It is characterized by aggressive and uncontrolled division of abnormal lymphocytes, which spread to various parts of the body and penetrate and destroy healthy body tissue [1]. Rapid identification and classification of the pathogenic cells is important for choosing the correct therapy for ALL patients. Conventional diagnosis comprises a combination of methods, including morphologic, cytochemical, cytogenetic, or immunologic tests [2, 3], as well as bone marrow biopsy [4]. Additional techniques to further classify the type of leukemia include flow cytometric immunophenotyping [5] and polymerase chain reaction studies [4, 6, 7]. A novel approach that may render these elaborate and invasive procedures unnecessary is based on the recognition of cancer-specific biomarkers on the surface of cancer cells by DNA/RNA aptamers [8].

Aptamers are synthetic short single-stranded DNA or RNA oligonucleotides that fold into unique three-dimensional shapes. These structures enable highly selective and specific targeting of molecules with high affinities comparable to those of antibodies. The small size and rather simple structure of aptamers relative to antibodies makes them easier to be synthesized and chemically modified. Moreover, they display low to no immunogenicity among other advantages. Therefore, aptamers have emerged as a new molecular tool in clinical medicine to detect and isolate proteins, and to act as targeting and therapeutic agents [9–12]. The DNA aptamer sequence sgc8c has been synthesized to specifically recognize ALL T-cells [13], where it is known to bind with high affinity (K_d_ = 0.8 ± 0.09 nM) to the protein tyrosine kinase-7 (PTK7) [14, 15]. PTK7 has also been found to be overexpressed in various other cancer types, including colorectal cancer and cancers of the lung, prostate, lymph nodes, and breast [16–19]. Thus, sgc8c has become a promising conjugate for targeted delivery of chemotherapeutics [20–22], photothermal agents [23, 24], immunotherapeutics [25], and contrast agents [26–28], and for noninvasive diagnosis [29] of cancer.

Recently, O’Donoghue et al. addressed the first step of sgc8c mediated cancer cell targeting on a single aptamer-receptor level using atomic force microscopy (AFM) [30]. The aptamer was linked to the tip of the AFM cantilever and brought into contact with the plasma membrane of HeLa cells. In their proof of principle experiment rupture forces of 46 ± 26 pN between sgc8c and PTK7 on the cell surface were measured only at one given force load and showed that the binding strength of aptamer and antibody to cancer cells was about equal under these setting. Here, we expand on this work, and include dynamic aspects of the molecular recognition between sgc8c and PTK7 on Jurkat T-cells by conducting single molecule force spectroscopy (SMFS) under variation of the force load. We performed AFM recognition imaging to gain data on the distribution of PTK7 receptors on Jurkat cells. SMFS has become an increasingly popular technique in the development of new pharmaceuticals to explore the interaction of new therapeutic molecules with cell membranes and whole cells [31–33]. The technique enables determination of energetic, thermodynamic, and kinetic parameters that describe the free-energy landscape of the interacting ligand target molecule complex [34, 35]. In particular, SMFS yields the dissociation rate constant (k_off_) and the width of the interaction potential (x_β_), which characterize the microscopic basis of bond formation of a ligand–receptor pair [36]. In this study we employed SMFS to measure the relative off-rate, the main determinant of the affinity between sgc8c aptamer and PTK7 receptor complexes. In addition, biosensing AFM also enables localization of binding sites and their distribution on cellular surfaces by using ligand-functionalized tips in analogy to those used in SMFS for high-resolution AFM imaging [31, 37, 38]. Here, we employ the well-established method of simultaneous topographic and recognition imaging (TREC), which has been successfully applied to localize binding sites on isolated molecules [39–42], artificial [43], and native membranes [44], as well as whole cells [45, 46] in the past decade. We performed AFM recognition imaging to probe the distribution of PTK7 receptors for the first time on the single receptor level on lymphoma cells.

## Materials and methods

All chemicals were used in their highest available purity. PBS and HBSS buffer salts, acetic acid, and citric acid were obtained from Sigma-Aldrich (Vienna, Austria), cell media and HEPES buffer were purchased from PAA (Pasching, Austria), and Cell-Tak from BD Biosciences (Erembodegem, Belgium). In all experiments only ultrapure MilliQ (MQ) water (Millipore, Darmstadt, Germany) with 18 MΩ resistance was used.

### T-cell culture and preparation

TIB 152 cells (Jurkat clone E6-1, ATCC, Wesel, Germany) were cultured in RPMI 1640 medium containing 10% fetal calf serum (FBS) supplemented with 1% penicillin/streptomycin and 1% HEPES buffer and maintained in an incubator under air atmosphere with 5% CO_2_ at 37 °C. Cells were passaged twice a week and reseeded at a concentration of 1:5. For AFM experiments, cells were used 3–4 d after splitting and immobilized on round glass slides (diameter 22 mm, VWR, Vienna, Austria). For cell attachment, glass slides were first cleaned with 80% isopropanol and ultrapure sterile MQ water (Millipore, Darmstadt, Germany) and dried. Next, the surface was coated with BD Cell-Tak in 5% acetic acid (Sigma-Aldrich) by hand-spreading the solution with a 50 μL glass micropipette and left for air drying under the laminar flow. After rinsing with 70% ethanol and a final washing step with sterile MQ water, cells were added. For this the cells were centrifuged and the cell pellet resuspended in 1500 μL RPMI media without FBS, supplements, and phenol red. Five hundred μL of cell suspension was transferred to each Cell-Tak coated glass slide and incubated under air atmosphere with 5% CO_2_ at 37 °C for 30 min. Cell density, their condition, and adherence were checked under the light microscope. Before chemical fixation, cells were rinsed three times with PBS buffer (137 mM NaCl, 2.7 mM KCl, 10 mM Na_2_HPO_4_, 1.8 mM NaH_2_PO_4_, pH 7.4) to remove media components, then treated with 4% formaldehyde in HBSS (Vienna, Sigma Aldrich) over a period of 60 min at room temperature, washed again in PBS three times, and used immediately or stored in the fridge for a maximum of 5 d.

### Preparation of sgc8c aptamer AFM sensors

The sequence of the PTK7 specific DNA aptamer sgc8c was: 5′ (SH- or) NH2- ATC TAA CTG CTG CGC CGC GAA AAT ACT GTA CGG TTA GA-3′. For the specificity proof the nonspecific DNA aptamers TDO5 was used: 5′ NH2- CAC CGG GAG GAT AGT TCG GTG GCT GTT CAG GGT CTC CTC CCG GTG-3′. The sequences of the aptamers were taken from the paper by Huang et al. [20]. Both aptamers were purchased from Thermo Fischer Scientific GmbH (Darmstadt, Germany). All aptamer solutions were prepared by dissolving lyophilized oligonucleotides in TE buffer (1 mM EDTA, 10 mM Tris, pH 8).

TREC measurements were performed with magnetically coated cantilevers MACLever Type VII (Keysight, Santa Rosa, USA). SMFS experiments were done with MSCT probes (Bruker, Karlsruhe, Germany). For both TREC and SMFS experiments the identical cantilever functionalization protocols were applied in which DNA aptamers were tethered to the apex of silicon(nitride) tips using a distensible heterobifunctional poly(ethylene) glycol linker. As silicon (nitride) is known to be an inert material, the tip was first chemically activated by deposition of APTES from the gas phase to ensure a sufficient number of reactive sites, but without forming a 3D network of the functionalization agent that would cause variations in the unbinding length of rupture events. For this, silicon (MACLevers) or silicon-nitride (MSCT) tips, respectively, were amino-functionalized according to the gas-phase deposition protocol published previously [47]. In brief, chloroform-cleaned cantilevers were placed in an argon filled 5 L desiccator together with a vial filled with 60 μL freshly distilled amino-propyl-triethoxysilane (APTES, Sigma-Aldrich, Vienna, Austria) and another vial with 20 mL trimethylamine (TEA, Sigma-Aldrich, Vienna, Austria), and allowed to react for 120 min. Then the desiccator was flushed with argon gas for 5 min and left for 48 h for curing process. For tethering the aptamers, either the NHS-PEG-acetal linker [48] was employed to covalently couple amine terminated aptamer NH_2_-sgc8c to the AFM tip or the NHS-PEG-PDP linker [49] to bind thiol terminated aptamer SH-sgc8c. Attachment of the reference aptamer TDO5 was done analogically.

In the case of NH_2_-terminated aptamers, freshly APTES coated tips were incubated in a chamber containing a solution of 1 mg NHS-PEG-acetal linker dissolved in 500 μL chloroform. Thirty μL TEA was added as catalyst. After 120 min reaction time, tips were washed with chloroform and ethanol (three times for 5 min.) and dried again in a gentle nitrogen gas stream. To obtain the aldehyde function, tips were immersed for 10 min in 1% citric acid solution, washed three times in water, and dried under N_2_ gas. The aldehyde functionalized tips were immersed in ~40 μL PBS solution containing 1 μM NH_2_-sgc8c aptamer and 2 μL freshly prepared aqueous solution of 1 M NaCNBH_3_ (Sigma-Aldrich, Vienna, Austria) was added to the drop, mixed carefully, and allowed to react for 1 h. Ten min before washing the tips, 5 μL of 1 M ethanolamine in water was added to the solution in order to passivate unreacted aldehyde groups. Finally, the tips were washed three times with PBS buffer and stored in PBS buffer at 4 °C until use.

Alternatively, coupling via the thiol residue of SH-sgc8c was done as follows. After APTES silanization as described before, cantilevers were in a solution of 1 mg NHS-PEG-PDP dissolved in 500 μL chloroform to which 30 μL TEA were added, and the cantilevers were allowed to react for 120 min. Subsequently, they were washed with chloroform and dried gently by N_2_ gas. Then the PDP functionalized tips were immersed in an SH_2_-sgc8c aptamer solution (10 μM in PBS) for 1 h, washed three times with PBS buffer, and used immediately or stored in PBS buffer at 4 °C for use within 5 d.

### Single molecule force spectroscopy (SMFS)

SMFS experiments were conducted on a PicoPlus 5500 AFM setup (Keysight, Santa Rosa, USA) equipped with a fluid chamber to allow measurements in PBS, and an optical CCD camera to facilitate cantilever alignment with immobilized T-cells on the substrate. Pulling experiments were performed in PBS using sgc8c functionalized MSCT cantilevers with nominal spring constants between 0.01 and 0.03 N m^−1^ under variation of the pulling velocity to yield loading rates (i.e., product of pulling velocity and effective spring constant) ranging from ~400 to 10^5^ pN s^−1^. The maximum indentation force was set to 500 pN to avoid any damage to the cells. At each pulling velocity, 1000 to 2000 force distance cycles (FDCs) were performed. To ensure position-independent results, the position on the cell was shifted by 250 nm μm after 250 FDCs. The delay between approaching and retraction period (i.e., the hold time) was varied from 0 to 1 s to ensure sufficient contact time for ligand–receptor bond formation.

The spring constant of each cantilever was determined according to the thermal noise method [50]. Statistical analysis of all FDCs were done to determine the probability of aptamer–receptor complex formation (binding probability, BP), the distribution of detected unbinding forces and lengths, as well as the effective spring constant (spring constant in the moment of rupture). The BP is defined as the number of FDCs exhibiting an unbinding event divided by the total number of collected FDCs. Each individual rupture force of a single unbinding event was plotted against its individual corresponding force loading rate r (determined from the effective spring constant multiplied by the pulling velocity) and finally merged into a dynamic force spectra plot. The loading rate-dependent unbinding forces were evaluated with a maximum likelihood approach [51] to fit a statistical model based on the Evans theory [52] that allows calculation of the dissociation rate constant (k_off_) and the width of the energy barrier x_β_ based on the equation f_u_(r) = (k_B_T/x)ln[rx/(k_B_Tk_off_)], where x is the separation of the energetic barrier to the equilibrium position, k_off_ the dissociation constant at zero force, k_B_T the thermal energy, and f_u_(r) the most probable unbinding force at the loading rate r.

### Simultaneous topography and recognition imaging (TREC)

In TREC magnetically coated and ligand functionalized cantilevers are excited by an alternating magnetic field to oscillate close to their resonance during scanning. The resonance frequency of biofunctionalized MACLevers Type VII was determined by recording a frequency plot, and the actuation frequency was typically set about 0.5 kHz below the maximum of the resonance. Then the cantilever was positioned above the T-cell of interest and slowly approached to avoid damage of the tip coating and/or the cell. Since the cells appeared very soft, recognition imaging was only performed on the outer area of the cells (i.e., the area of the cells that have the higher distance to the middle of the cell). TREC measurements were done in PBS buffer at 1 Hz line frequency. The amplitude was set to a value obtained by force distance cycles, which ensures sufficient damping of the upper part of the oscillation in case of molecular (sgc8c–PTK7) interaction, but at the same time allows remaining in a bound state while measuring above the recognition site [53]. All other measurement parameters were identical to the SMFS experiments.

For data evaluation, the threshold of recognition spots was set to five times the root mean square (rms) of the flattened recognition image. The spot size for recognition was set to a typical value of 15 pixels for a 1 × 1 μm scan at 512 pixel/line. A mask overlay of the determined recognition spots with the corresponding topographic image was generated using Gwyddion FreeSPM software (ver. 2.44).

## Results and discussion

**Fig. 1:**
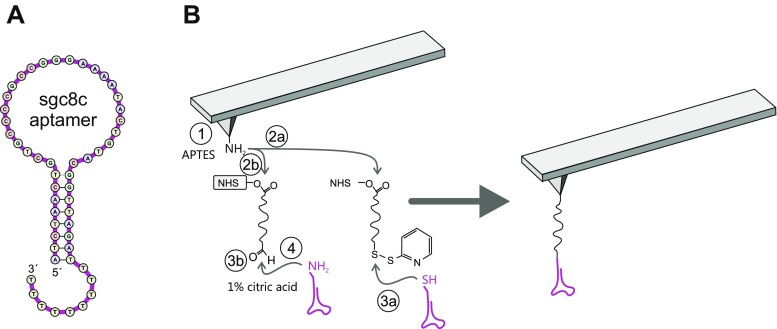
(**A**) DNA sequence of the used sgc8c aptamer. (**B**) Tip chemistry. (1) Inert silicon nitride cantilever are amino-functionalized using APTES gas phase silanization. The heterobifunctional crosslinker NHS-PEG-PDP (2a) or NHS-PEG-Acetal (2b) is coupled allowing binding NH_2_-terminated (4) DNA aptamers [after deprotection of the acetal group (3b)] or SH-terminated sgc8c aptamers (3a) respectively

The DNA aptamer sgc8c (Fig. 1A) has been designed to specifically recognize the receptor PTK7 in the plasma membrane. It is an important player in extracellular signaling and highly expressed on the surface of leukemia cells and especially T-ALL cells. In this study, we used cultured T-cells of the Jurkat non-Hodgkin’s lymphoblastic leukemia cell line as an accurate representation of the native state of PTK7 in lymphoma cells.

**Fig. 2:**
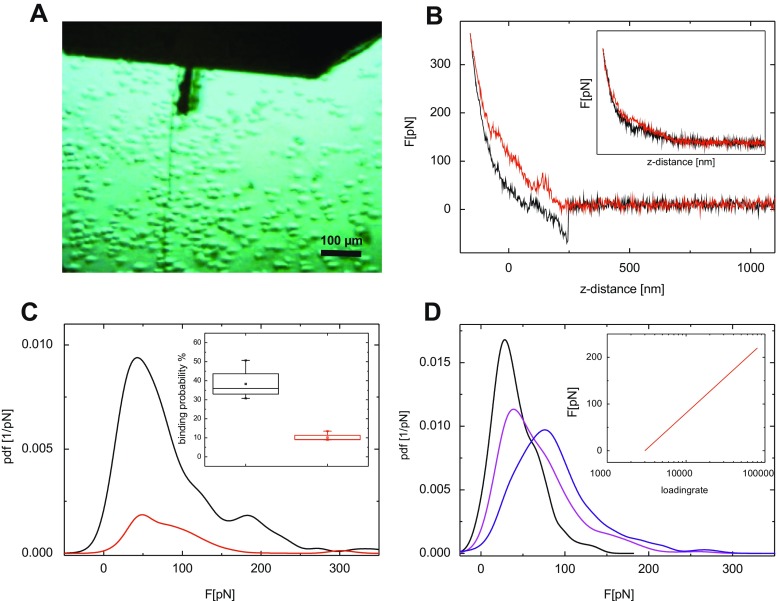
SMFS experiments: (**A**) Optical Image of T-cells on Cell-Tak coated glass slide. The bio-functionalized cantilever (upper middle) is placed above a single cell before approaching. (**B**) Typical force distance cycle using a sgc8c functionalized tip on a T-cell. In the retraction part (black line) a downwards bending is observable as a result of specific interaction. In contrast, in the presence of free sgc8c this interaction is blocked (inset). (**C**) Typical probability density function before (black) and after (red) addition of free ligand. The red curve is normalized to the relative binding probability. The significance of the proof experiment is shown in the inset. (**D**) Probability density functions of sgc8c–T-cell interactions at different pulling velocities. The most probable unbinding force increases at higher pulling velocities. The fit of the resulting loading rate dependence of the rupture force is shown in the inset

**Fig. 3:**
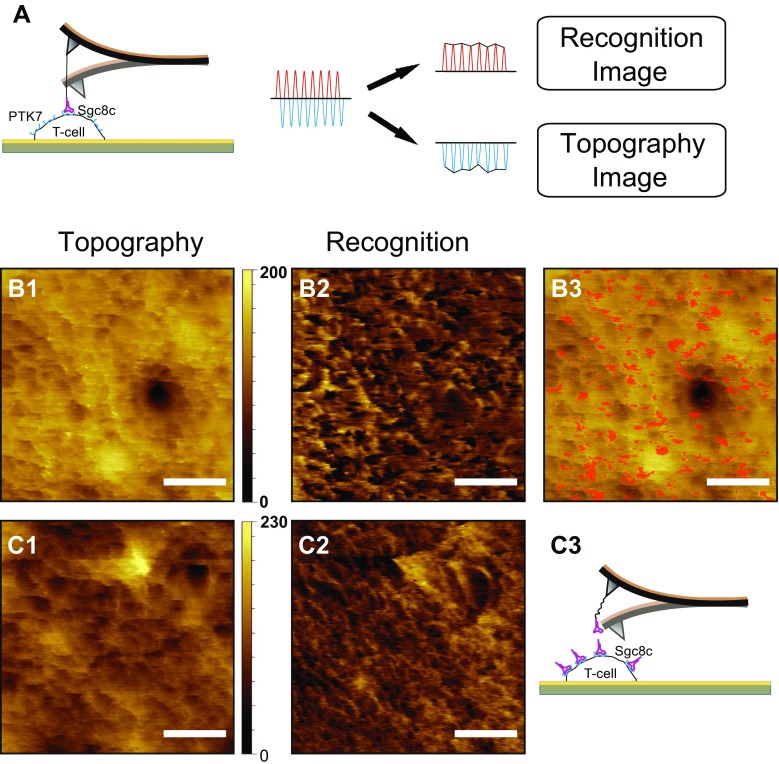
TREC experiments: (**A**) Schematic of TREC setup. The upper part of the oscillation is used to gain the recognition image, whereas the lower part is influenced by the sample topography. Topography (**B1**) and simultaneously acquired recognition (**B2**) image on a T-cell membrane using sgc8c functionalized tips. A superposition of topography and recognition is shown at image (**B3**). After addition of free aptamers the topography (**C1**) remains unchanged, whereas the recognition spots (**C2**) is completely abolished as a result of blocked PTK7 receptors (illustrated in **C3**). Scale bar for all AFM images is 500 nm

For characterization of the specific interaction of sgc8c with PTK7 on the cell surface at the molecular level by SMFS, the DNA aptamer was chemically attached to the apex of the silicon (nitride) tip of the AFM cantilever via heterobifunctional crosslinkers (Fig. 1B). After aminofunctionalization (Fig. 1B-1), depending on the coupling group of the aptamer, which was either 5′aminofunctionalized (NH_2_-sgc8c) or 5′ thiolated (SH_2_-sgc8c), an NHS-PEG-acetal [48] or an NHS-PEG-PDP [49] was covalently bound to the tip, respectively (Fig. 1B-2a, 2b). Thiolated sgc8c was coupled without further derivatisation simply through incubation to the PDP terminus of the tip-bound linker (Fig. 1B-3a). Binding of NH_2_-sgc8c required de-protection of the free acetal end of the linker to produce an aldehyde residue (Fig. 1B-3b) for reaction with the amine terminus of the aptamer followed by hydration of the formed bond after coupling.

T-cells, like all lymphocytes, circulate in the blood stream without adhesion to blood and lymphatic vessels, and adhesion and migration through the wall of vessels usually happens only in response to inflammation. Thus, Cell-Tak was used as adhesive coating to immobilize T-cells on glass cover slips for AFM investigation [54]. The cell suspension was handled carefully to avoid cell lysate of broken cells that may inactivate Cell-Tak and result in inefficient adherence of intact T-cells. Next, cells were chemically fixed. This was necessary since T-cell adhesion as the first step in immune response is known to start a cascade of changes of the cellular state that include rolling, cell arrest, strengthening of adhesion sites, followed by migration [55]. Incubation with 4% formaldehyde for 60 min was performed, which has been reported to properly fix cells without loss of functionality of PTK7 receptors on the plasma membrane [56].

For AFM force spectroscopy measurements, the sgc8c functionalized cantilever tips were positioned above a fixed cell using the optics of the AFM setup (Fig. 2A) and approach-retract cycles were executed, and the acting force on the cantilever was monitored as depicted in Fig. 2B in the form of a force-distance curve (FDC). In the approach-phase (Fig. 2B [red curve]) the cantilever starts to experience an increasing force and bend upward upon contact with the cell. The nonlinear slope of the first part of bending is a result of partly compressing of the cell. After reaching a given indentation force limit of typically 300–400 pN, the retraction phase was initialized (Fig. 2B [black curve]). In case sgc8c had formed a complex with PTK7 on the cell surface, further retraction of the cantilever lead to a pulling force, which appeared as negative slope in the retract part force due to a downward bending of the cantilever. When the pulling force exceeded the binding strength between the aptamer on the tip and PTK7 receptor on the Jurkat cells, a clear rupture event was observed with a rupture force corresponding to the sgc8c-PTK7 bond strength.

For statistical analysis, 500–2000 FDCs were recorded under variation of the location on the cell surface to avoid measuring artefact. To differentiate these supposed specific rupture events from nonspecific adhesion, and to avoid measuring artefact FDCs were collected on different positions on the cells and the hold time (i.e., the resting or hold time of the functionalized cantilever on the cell surface) was varied from 0 to 1000 ms, with no observable difference for the number or appearance of rupture events or measured rupture forces. In a few cases, double or multiple rupture events were observed. Such events are either caused by the binding of additional tip tethered sgc8c molecules to further PTK7 receptors on the cell or by nonspecific adhesion of the tip with the cell. Nonetheless, they were not used for the data evaluation since differentiation of these events cannot be done with sufficient accuracy.

To prove the specificity of the detected rupture events, the experiment was performed in the presence of free sgc8c aptamers in solution (1 μM) to saturate (block) PTK7 receptors on the cell surface. This resulted in a significantly lowered binding probability. In Fig. 2C the distribution of measured rupture forces is plotted in the form of a mathematical probability density function (pdf) before (black) and after (red) the addition of the blocking is shown for a representative cell. The red curve is normalized to the relative binding probability. The existence of a remnant BP may be explained by an incomplete block of accessible PTK7 receptors on the cell surface. The averaged binding probability of 38.3 ± 7.48% before the block was reduced to 10.15 ± 1.93% after addition of sgc8c to the Jurkat cells (Fig. 2C, inset). An additional, complementing specificity proof was performed with a tip that was functionalized with the TDO5 DNA aptamer that recognizes Ramos cell of the Burkitt’s lymphoma type, but does not bind to PTK7. The binding probability was only 8.27 ± 1.53%.

The rupture force of a ligand–receptor complex is dependent on the kinetics of the experiment, in particular the force ramp of the pulling force acting on the complex during rupture, and is termed the loading rate [52, 57]. From extrapolation of the rupture force dependence on the loading rate, the dissociation rate constant k_off_ and the width of the interaction potential x_β_ of the sgc8c-PTK7 interaction on the cell surface can be calculated. Experimentally, the loading rate is the product of the pulling speed and the effective spring constant at the point of rupture. Hence, variation of the loading rate is achieved by a change of pulling speed of the cantilever. The effective spring, on the other hand, is determined from a fit of the slope of the force curve at the point. Datasets were measured at pulling speeds ranging from 500 to 24000 nm s^−1^ and the rupture forces, rupture lengths, and effective spring constants for each single rupture event were identified. Statistical analysis at a given pulling speed showed that the increase of the pulling velocity caused an increase of the rupture forces, as illustrated by a shift of the maximum of the pdf (Fig. 2D). This is in good agreement with Evans’ theory for a single energy barrier in the ligand–receptor interaction potential in the thermally activated regime [52]. For calculation of k_off_ and x_β_ from the loading rate dependence, all individual rupture events were plotted as a data cloud to account for the influence of the effective spring constant that varies strongly at a given pulling velocity as a result of the position-dependent elasticity of the cell [58]. A fit of the data by a maximum likelihood approach (as described in more detail in [51]) yielded a dissociation constant k_off_ of 5.16 ± 0.19 s^−1^ with a width of the energy barrier x_β_ of 0.65 ± 0.01 Å. The kinetic off rate is indicative of a slow dissociation of the complex, which is favorable in terms of an extended interacting time between the aptamer and the PTK7 in the cell membrane.

In SMFS the functionalized AFM cantilever probes the surface “blindly,” which has an influence on the probability to form ligand–receptor complexes to measure their rupture. Whereas the binding probability on a dense layer of isolated receptors can be as high as 70 to 80%, binding probabilities on cells range typically below 20% due to a less dense and less homogenous distribution of receptors and possibly preferential location in more specified membrane domains [59]. In the presented study, an uncommonly high average binding probability of nearly 40% was detected, leading us to investigate the distribution of PTK7 on the surface of Jurkat T-cells more closely by TREC.

The method of TREC is illustrated in Fig. 3A; a magnetically coated cantilever with a quality factor in liquid of ~1 carries an sgc8c aptamer tethered to the tip via a flexible linker. The tip oscillates above the cell surface driven by an alternating magnetic field (MACmode). During lateral scan at a rate of 0.5–1.0 Hz, sgc8c can bind to PTK7 in the downward swing of the oscillation, which leads to linker stretching and reduction of the upward swing. Separation of the bottom part of the oscillation amplitude that contains the topographic information from the top part that is only influenced by binding events, a topography image and recognition map are created from a single scan with lateral resolution of a few nanometer [53]. To provide for sufficient contact time that enables ligand–receptor complex formation and in order to achieve high lateral resolution, surface scans were performed at 1.5 × 1.5 μm^2^ scan range (Fig 3B). However, due to the very soft behavior and high compressibility of the gently fixed spherical T-cells, TREC imaging on top of a cell turned out to be more challenging. Hence, all images displayed in Fig. 3 were captured towards the border area of a cell. The topography revealed a rather smooth surface (Fig. 3B-1, C-1) but a high number of pronounced dark patches (recognition spots) that reflect positions of aptamer binding sites on the Jurkat cell (Fig. 3B-2). Figure 3B-3 shows the overlay of the recognition events indicated in red with the topography image and show that PTK7 was evenly distributed over the scan area. In order to prove that the detected aptamer binding sites were indeed the locations of PTK7 receptor molecules sgc8c saturated T-cells were scanned using the identical AFM tips with the same imaging parameters and settings [53]. Whereas the topography images showed the same features before (Fig. 3B-1) and after block (Fig. 3C-1), the recognition signals were nearly completely abolished (Fig. 3C-2) due to block of the PTK7 receptors by the added sgc8c DNA aptamers, as illustrated by the cartoon in Fig. 3C-3, proving that the recognition image in 3B2 shows the distribution of PTK7 in the cell membrane. The TREC imaging revealed a homogeneous distribution of PTK7 molecules on the outer regions of ALL cells with a surface density of 325 ± 12 PTK7 receptors (or small receptor clusters) per μm^2^.

## Conclusions

In this study, we demonstrated the specificity of the interaction of the sgc8c DNA aptamer and PTK7 receptor in the plasma membrane of intact Jurkat T-cell lymphoma, and measured rupture forces of the ligand–receptor complex under different loading rates using SMFS. The kinetic off rate of the studied system (k_off_ = 5.16 s^−1^) indicates slow dissociation of the complex. Furthermore, we demonstrated the possibility of performing recognition imaging experiments on T-cells for the first time. TREC represents a powerful imaging tool in which topography and recognition of specific biological molecules are simultaneously mapped. Our results show that aptamers covalently coupled to an AFM probe recognize specific receptors at nanometer lateral resolution and with high specificity. More importantly, we were able to visualize and quantify the distribution of PTK7 receptors in the cell membrane, showing a high density and homogenous lateral distribution in ALL-cells, making it an ideal target. This study provides new insight into the mode of the action of the sgc8c DNA aptamers as a diagnostic and targeting agent for acute lymphoblastic leukemia. As the continuing advancements of the cell SELEX technique [60] yield a growing library of aptamers for various cancer markers, they may prove particularly useful for identification and capturing of circulating tumor cells (CTC) [61]. Moreover, our study presents validation of the method of AFM biosensing for the detection of cancer markers and reveals potential for future clinical diagnostics. In combination with traditional histology-based analysis of biopsies, the method could reduce tumor misclassification [62]. Also, with the expression level of tumor markers being related to the clinical stage of the disease, determination of the marker density on the cell surface by TREC might allow for assessment of disease progression as well as the efficacy of cancer therapies.
